# Comparison of digital and visual tooth shade selection

**DOI:** 10.1002/cre2.721

**Published:** 2023-02-13

**Authors:** Sabrin Abu‐Hossin, Yonca Onbasi, Lara Berger, Florian Troll, Werner Adler, Manfred Wichmann, Ragai‐Edward Matta

**Affiliations:** ^1^ Department of Prosthodontics Erlangen University Hospital Erlangen Germany; ^2^ Department of Medical Informatics, Biometry and Epidemiology Friedrich‐Alexander‐University of Erlangen‐Nuremberg Erlangen Germany

**Keywords:** Cerec Omnicam, intraoral scanner, shade selection, tooth color, Trios 3, visual shade selection

## Abstract

**Objective:**

In dental restorations, color determination is very important for achieving esthetic results. The aim of this study was to compare visual shade selection using digital methods and to assess the repeatability of the utilized intraoral scanners.

**Materials and methods:**

In 31 probands, tooth color was determined on teeth 11, 13, and 16. Shade selection was performed visually by a dentist and digitally using Trios 3 and Cerec Omnicam. Three measurements were performed to determine the repeatability of intraoral scanners. Fleiss' *κ* was used for statistical evaluation of the repeatability and Cohen's *κ* was used for comparison of methods.

**Results:**

The visual method showed only slight agreement with Trios 3 (Cohen's *κ*: 0.198) and Cerec Omnicam (Cohen's *κ*: 0.115). Moderate agreement was found between Trios 3 and Cerec Omnicam (Cohen's *κ*: 0.452). In terms of repeatability, Trios 3 scored higher overall than Cerec Omnicam (Fleiss' *κ*: 0.612 vs. 0.474).

**Conclusion:**

Intraoral scanners can facilitate the workflow in clinical practice. They are a good supplement for color determination, but should additionally be confirmed by the visual method. Clinical significance: The use of digital instruments is increasingly being preferred over conventional treatments. Therefore, it is essential to continuously improve the accuracy of intraoral scanners for color selection in order to offer an alternative to visual methods.

## 1 INTRODUCTION

Esthetic factors are becoming increasingly important in modern dentistry. Dental restorations must accurately reproduce the exact tooth shade, matching the neighboring teeth, to ultimately satisfy the patient's esthetic demands (Bahannan, 2014; Ebeid et al., 2021; Gotfredsen et al., 2015; Mehl et al., 2017). However, human color perception is influenced by many different external and internal factors, such as brightness and saturation (Devigus, 2003; Liberato et al., 2019).

To achieve the best possible therapeutic result, suitable shade guides and dental materials must be available, and an experienced dentist should perform the shade taking and data transfer to the laboratory. Additionally, a qualified dental technician is required to accurately reproduce the determined tooth shade and incorporate it into the restoration (Paravina et al., 2001). From the patient's perspective, along with shape and position, color determination is among the most important aspects of dental treatment, in order to achieve a natural treatment result and ensure satisfaction with the new dental restoration (Brandt et al., 2017). Corrections or even new fabrications are associated with increased costs, and expenditure of time and effort for the technician, dentist, and patient. A dental prosthesis generally serves to restore lost teeth or to preserve individual teeth in need of crowning, such that it is critical to use the correct tooth shade, particularly in the anterior region of the maxilla (Reyes et al., 2019).

In addition to visual and spectrophotometric color determination using digital cameras and colorimeters, digital shade selection is also possible using various intraoral scanners (Czigola et al., 2021; Klotz et al., 2020; Liberato et al., 2019; Yoon et al., 2018) that are based on different technical backgrounds (Ebeid et al., 2021). The aim is to replace subjective observation by the human eye with precise and objective shade determination (Hampé‐Kautz et al., 2020). However, the most frequently used method in the dental sector is visual color determination (Culic et al., 2018; van der Burgt et al., 1990), which is usually based on standardized industrially specified color ring samples (Joiner, 2004; Rutkūnas et al., 2020). In this context, the shade rings of the manufacturer VITA, including the Vita Classical A1‐D4 and the VITA 3D‐Master (VITA Zahnfabrik), are well established in the dental market (Paravina et al., 2001; Rutkūnas et al., 2020).

Human color perception is based on complex physiology, as the emitted electrical signals are further processed and interpreted in the brain. These signals originate from different cells that are sensitive to specific ranges of the light spectrum (Reyes et al., 2019). However, visual color perception can be manipulated by subjective aspects, such as eye fatigue, work experience, or age, and by environmental factors, such as the surroundings, lighting conditions, and the metamerism phenomenon, creating potential sources of error (Brandt et al., 2017; Liberato et al., 2019; Paul et al., 2002; Reyes et al., 2019).

Notably, the physiological color recognition of all participants is assumed (Joiner, 2004), but the color choices of different observers do not always match (Devigus, 2003). Therefore, color determination should not depend on the day, age, or gender, and should always be reproducible (Berns, 2019; Paul et al., 2002). Despite the limitations of the visual method, the human eye is very accurate and precise in detecting the smallest color differences between two objects, such that visual perception is indispensable before the placement of a dental prosthesis (Gotfredsen et al., 2015; Paul et al., 2002).

Innovative developments in digital dentistry ensure the practitioner a digital workflow from diagnosis and planning to treatment and modeling of dental prothesis or digital smile design (Solaberrieta et al., 2016). The use of computer‐aided design (CAD) and computer‐aided manufacturing (CAM) technology has become indispensable in many dental practices and dental laboratories, and is especially used in prosthetic dentistry and orthodontics. The digital workflow enables increased patient comfort and easier practitioner–technician communication, and is associated with lower costs (Aeran et al., 2014).

Since 2017, intraoral scanners have included software for shade selection, which can be performed following an intraoral scan (Culic et al., 2018). For example, Cerec Omnicam (Dentsply Sirona) and Trios 3 (3shape) include this function and can thus support the digital workflow while replacing visual color perception. Tooth color is determined by the interaction of a high‐resolution camera, an LED light, and computer software with the VITA shade guide as a reference (Gotfredsen et al., 2015; Moussaoui et al., 2018). Digital technologies can provide enormous advantages in everyday clinical practice and ensure more precise results in shade selection. Additionally, they can facilitate easier communication between the treating dentist and the dental technician, and better esthetic results (Culic et al., 2018; Moussaoui et al., 2018).

Correct shade selection can be affected by the texture of the natural teeth. For example, tooth color is determined by surface morphology, translucency, and background environment (Kim‐Pusateri et al., 2009). Different degrees of enamel translucency and opacity of the dentin may also complicate the shade selection, resulting in a shade being beyond the shade guide used (Kim‐Pusateri et al., 2009; Yoon et al., 2018).

In this clinical study, the aim was to compare visual shade determination by a dentist with the digital method and to investigate the repeatability of the intraoral scanners Trios 3 and Cerec Omnicam. However, there are controversial findings regarding the accuracy of the different methods of shade determination. The research hypotheses were as follows: (1) there is no difference between the visual and digital shade determination methods and (2) digital measurements of tooth color are always repeatable and provide the same in color selection results.

## 2 MATERIALS AND METHODS

In this clinical study, the aim was to comparatively evaluate the digital method of tooth shade determination and the commonly used visual method of shade selection. The project was approved in advance by the Medical Ethics Committee of the Friedrich‐Alexander University Erlangen‐Nuremberg (approval number: 416_18B) and was registered in the German Register for Clinical Studies (reference number: DRKS00027988).

The volunteer patient collective was selected at the Dental Clinic 2—Dental Prosthodontics of the Erlangen University Hospital of the Friedrich‐Alexander University of Erlangen‐Nuremberg. The following gender‐independent inclusion criteria were applied for study participation: no periodontally damaged or deeply destroyed teeth, no prosthetic restorations, no mineralization disorders, good oral hygiene, and age of majority (≥18 years). After signing the subject consent form, a total of 31 patients were enrolled in the study. The study participants' ages ranged from 22 to 32 years.

### 2.1 Procedure for shade determination

In the course of visual shade determination, all the teeth of each subject were initially examined for cleanliness and cleaned if necessary. To exclude external influences as much as possible during the examinations, color determination was conducted simultaneously in three neighboring treatment units, during the same time window between 10 a.m. and 1 p.m. Additionally, the lamps in the units were switched off to ensure that the ambient lighting was as identical as possible and so that natural daylight was available. To avoid high‐contrast impressions and potential manipulation of the results, the subjects were asked to wear discreet makeup and the outer clothing of all study participants was covered with a patient napkin. Then, an experienced dentist performed the actual shade determination on the vestibular surfaces of upper right central incisor 11, canine 13, and first molar 16 using the commonly applied VITA Classical A1‐D4 shade guide (VITA Zahnfabrik). This comprises 16 tooth shades, which can be divided into four groups according to shade (A, B, C, and D) and then further subdivided based on intensity (i.e., brightness) and saturation (1, 2, 3, and 4) (Bratner et al., 2020).

To avoid influencing the visual method, another experienced dentist performed the digital shade determination using the Trios 3 (3shape) and Cerec Omnicam (Dentsply Sirona) intraoral scanners, which had been calibrated in advance following the manufacturer's instructions. During digital color determination, teeth 11 and 13 were each divided into cervical, middle, and incisal sections to enable measurement of as much of the same area of the teeth as possible. The tooth shade was then registered for the middle third, since the shades of the other two regions can be influenced by the gingiva in the cervical section and by the increasing transparency in the incisal tooth area (Gotfredsen et al., 2015; O'Brien et al., 1997). In contrast, for molar 16, the mesiobuccal area was used for color determination. To check the repeatability of the individual color measurements of both scanners, three color measurements were performed for each tooth.

After proper tabular documentation of the measured values, statistical evaluation was performed. Cohen's *κ* was calculated for pairwise comparison of the shade acquisitions of the dentist, Cerec Omnicam, and Trios 3. Fleiss' *κ* was used to evaluate the repeatability of the individual scanners (Table 1).

**Table 1:** Interpretation of the Kappa statistic.

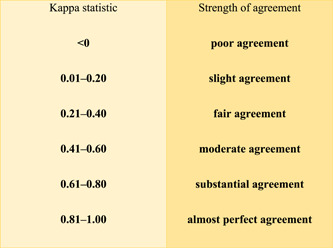

## 3 RESULTS

**Figure 1:**
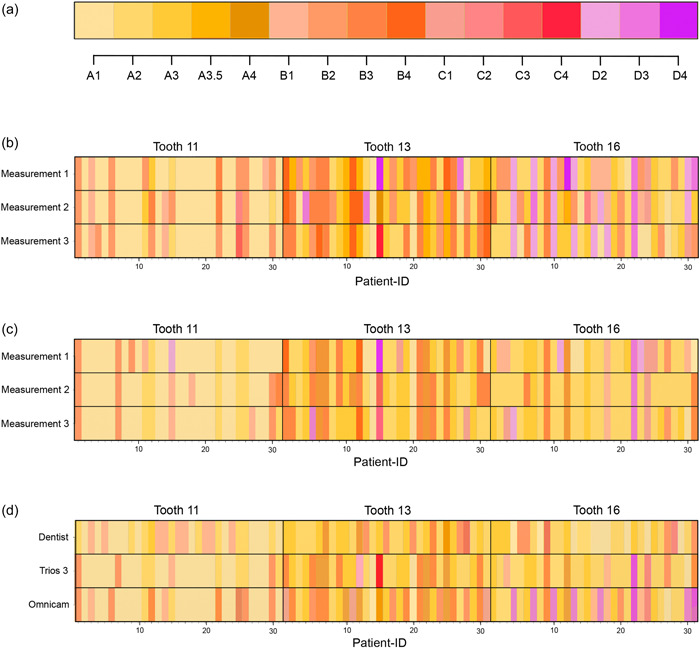
Results of the repeatability of the individual color measurements by the two scanners Cerec Omnicam (b) and Trios 3 (c) using 3 measurement repetitions for each tooth. In addition, comparison was performed of the different shade determination methods (d) following statistical majority decision of the respective measurement repetitions carried out by the intraoral scanners applying the corresponding color scale (a) for all measurements.

Analyses were performed using the results of the visual and the digital shade determinations for all 31 study participants (Figure 1). Cohen's *κ* was determined for comparison of the visual color measurements by the dentist with the digital color measurements and for comparison between the intraoral scanners. Cohen's *κ* was 0.198 for the overall comparison of the color measurements by the dentist versus Trios 3 and 0.115 for measurements by the dentist versus Cerec Omnicam. Comparison between the two digital methods revealed a higher agreement (Cohen's *κ*: 0.452). When considering only the values for the determined shade, the overall Cohen's *κ* for all comparisons among the different systems was smaller, indicating weaker agreement between the measurements than between the intensity shade values (Table 2).

**Table 2:** Cohen's *κ* values for the respective pairwise comparisons between the visual system, Trios 3, and the Cerec Omnicam as a whole, for the abstracted shade, and for the intensity.

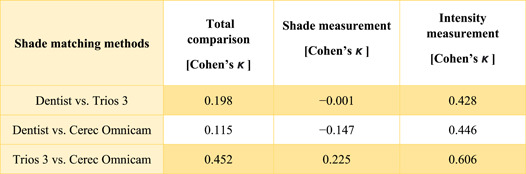

The overall repeatability of the digital shade determinations was found to be higher with Trios 3 compared to with Cerec Omnicam (Fleiss' *κ*: 0.612 vs. 0.474). Considering the separate measurements of each tooth, the repeated shade determinations of tooth 11 showed the highest agreement with both Trios 3 and Cerec Omnicam (Fleiss' *κ*: 0.688 vs. 0.594) compared to teeth 13 and 16.

Furthermore, the repeatability of the respective indicated shade (A–D) as well as the intensity (1–4), with both intraoral scanners, were separately calculated to further investigate differences and thus the repeatability of the utilized digital systems. For the shade measurements, there was moderate overall agreement for both Trios 3 and Cerec Omnicam (Fleiss' *κ*: 0.469 vs. 0.412). In contrast, substantial agreement for the intensity measurements of all teeth was found (Fleiss' *κ*: 0.676 vs. 0.618) (Table 3).

**Table 3:** The overall repeatability and repeatability of the indicated shade and intensity when using Trios 3 and Cerec Omnicam for the colorimetric repeats of all three teeth examined and separately for teeth 11, 13, and 16.

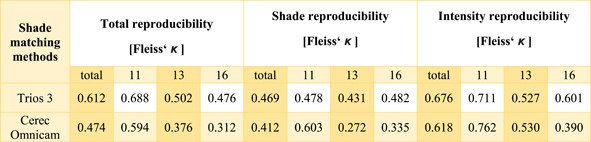

## 4 DISCUSSION

Digital intraoral scanners are playing an increasingly important role in color determination in dentistry (Czigola et al., 2021; Liberato et al., 2019; Rutkūnas et al., 2020). High‐precision digital color determination could facilitate the workflow in everyday dentistry and replace the conventional visual method (Brandt et al., 2017; Moussaoui et al., 2018). Literature research reveals a lack of investigation of different intraoral scanners with regard to color determination, even though they play an important role in clinical application. Trios from 3shape is considered a pioneer in the development of color determination software, and several studies investigate this tool in comparison with the visual method and/or with the spectrophotometer (Brandt et al., 2017; Gotfredsen et al., 2015; Mehl et al., 2017). The results of the present study did not confirm the first research hypothesis “There is no difference between the methods of visual and digital color determination.”

A previous systematic review identified only three studies evaluating the accuracy, repeatability, and reproducibility of intraoral scanners in digital shade determination (Moussaoui et al., 2018). Comparisons revealed no significant difference between shade determination with Trios from 3shape versus visual shade determination in terms of accuracy and repeatability. Despite differences in the study designs, the authors propose that intraoral scanners are a good alternative to conventional visual determination (Moussaoui et al., 2018).

In an in vivo study of 87 teeth from 29 patients, the reliability of the objective method was very high compared with visual color determination (considerable agreement, with *κ* values of 0.61 and 0.72 for two practitioners), and no significant differences were found between the Trios Color System and the visual method (Gotfredsen et al., 2015). On the other hand, the present results comparing Trios 3 and Cerec Omnicam with visual color determination showed poor overall agreement (Cohen's *κ*0: 0.198 and 0.115).

Similar to this study, other investigations have used the middle third of the anterior teeth to determine the most accurate shade (Gotfredsen et al., 2015). This area provides the most reliable results when determining tooth shade, as the shade selection is not distorted by external influences. In contrast, shade selection can be influenced by translucency in the incisal third or by the proximity of the gingiva in the cervical third (Gotfredsen et al., 2015; O'Brien et al., 1997).

Comparison of the hue revealed a slight match. This can be explained by the frequent determination of shade A, in contrast to B, C, or D. These shades were determined for only a few teeth, and were thereby decisive for the Kappa value, which conversely turned out to be very low. Detailed literature research reveals that no prior study has divided tooth shade into hue and intensity and checked the accuracy and repeatability of these assessments.

Previous studies have primarily performed visual determination using the VITA Classical A1–D4 shade guide or the VITA Toothguide 3D‐Master (Gotfredsen et al., 2015; Hampé‐Kautz et al., 2020; Joiner, 2004; Liberato et al., 2019). In the present study, to evaluate the practical suitability of the utilized intraoral scanners, with regard to cost‐efficient and economical working due to possible time saving, the practitioner performed shade determination using the VITA Classical A1‐D4 shade guide, which is common and most frequently used in the clinic. Based on this, the color values determined using the software of the Trios 3 and the Cerec Omnicam were also displayed in the VITA Classical A1–D4 color scheme.

The present findings only partly confirmed the second research hypothesis, which concerns the repeatability of the utilized Trios 3 and Cerec Omnicam intraoral scanners, when shade determination was repeated three times. The results of this study showed substantial agreement in repeatability for Trios 3 (0.612) and moderate agreement for Cerec Omnicam (0.474).

In an in vivo study, visual color determination was compared with instrumental color determination, and the repeatability of Trios 3 was also tested. The results of that study underscore the data obtained in the present investigation. In a total of 28 test subjects, the tooth shade of the upper central incisor was determined, and the repeatability was assessed over three passes. Trios 3 achieved a substantial agreement, with a Fleiss' *κ* of 0.639, which is very close to the present result (0.688). Overall, the authors concluded that instrumental shade selection is more reliable than visual determination using VITA Classical A1–D4 and VITA Toothguide 3D‐Master. The discrepancies between the different shade determination methods highlight the difficulty of and problems associated with selecting the correct shade for a highly esthetic restoration (Liberato et al., 2019).

Another in vitro study also investigated the repeatability and reproducibility of intraoral scanners, including Trios 3 and Cerec Omnicam. In that study, color determination was carried out on ceramic blocks of 10 different shades from VITA Classical A1–D4 and repeated 10 times for each sample. The collected data and analysis showed worse than expected results in terms of accuracy (Trios 3 = 66%, Cerec Omnicam = 57%) and repeatability (Trios 3 = 51.7%, Cerec Omnicam = 57%), indicating that color determination should also be performed by an experienced practitioner as a supportive measure. In addition, although this difference was not statistically significant, the Cerec Omnicam showed the lowest accuracy when comparing the tested intraoral scanners, which could be related to lower scanning accuracy (Ebeid et al., 2021). In this regard, the results of that study are in overall agreement with the present findings.

Since color analysis software has been newly introduced in recent years, few studies have investigated the accuracy and repeatability of color determination, especially with Cerec Omnicam. In one study, the authors concluded that the Cerec Omnicam color analysis software—which is designed to speed up the overall clinical workflow—requires substantial improvements to achieve reliable color selection results (Culic et al., 2018). This result is supported by the present findings.

That previous study also demonstrated that the Cerec Omnicam should not be used as the sole method of color determination and that visual color determination should be performed to support digital color determination (Culic et al., 2018). Further research should be conducted to establish future advancements of these systems as facilitators in everyday clinical practice and to support their wider use.

Limitations of this study, as in other studies, included the lack of a time limit for the visual color measurement and the lack of a standardized scanning process. For example, this means that eye fatigue could not be considered. Other factors that can affect the color determination process include the scanning angle, direction of movement, scanning distance, the experience of the practitioner, and individual characteristics in the oral cavity (Liberato et al., 2019; Yoon et al., 2018). Another restriction was that the shade guide VITA Classical A1‐D4, which has only 16 shade tabs, was used. On the other hand, the shade guide of the VITA Toothguide 3D Master contains 28 shade tabs and enables more multifaceted determination of tooth shade by dividing it into brightness (value), color intensity (chroma), and shade (hue) in three steps (Gotfredsen et al., 2015).

Future studies should continue to test the latest digital technologies, such as the successors of Primescan and Trios 4, for accuracy and repeatability of shade selection. In this context, it should be investigated whether newer intraoral scanners show a technical improvement in color determination and may thereby adequately replace the visual method. Additionally, further in vivo studies are needed to test the color determination for the fabrication of restorative work and to evaluate the result in terms of esthetics and correct color selection once the denture is fitted. Comparisons should be made between intraoral scanners, spectrophotometers, and visual determination by a dental technician.

## 5 CONCLUSIONS

Within the limitations of this current study and based on the first research hypothesis, it was concluded that visual shade determination by an experienced practitioner remains extremely important and supports the use of digital intraoral scanners. Determining the correct color is a critical part of the fabrication of dentures or tooth reconstructions, as the smallest color differences can negatively influence esthetics.

Furthermore, based on the second research hypothesis, it was concluded that it remains of great importance to improve the technology of intraoral scanners and to increase the repeatability, with regard to color determination, so that practitioners rely more on digital methodology in the future.

## AUTHOR CONTRIBUTIONS

Sabrin Abu‐Hossin led the clinical investigations and was significantly involved in the manuscript preparation and the literature review. She also compared the results of the present literature with those obtained in our study. She also edited and created the illustration. Yonca Onbasi also performed the clinical examinations. She participated in the editing of the manuscript as well as in the analysis and evaluation of the results. Lara Berger helped with the conduction of the study and wrote part of the material and methods and also part of the results. Florian Troll was responsible for the coordination and documentation of the clinical investigation. In addition, he prepared an overview with the measured values for the statistician. Werner Adler is our statistician. He conducted all statistical tests and supported the interpretation of the results. Manfred Wichmann is director of the prosthetic department in Erlangen. His work experience and expert knowledge in the prosthetic field and in science, as well as permanent corrections, helped the authors in preparing the manuscript. Ragai‐Edward Matta is the project manager. He helped with the interpretation of the results and constantly made corrections to the manuscript. All authors have contributed to the submitted manuscript and co‐authored it.

## CONFLICT OF INTEREST STATEMENT

The authors declare no conflict of interest.

## Data Availability

The data that support the findings of this study are available from the corresponding author upon reasonable request.
